# IL-18和IL-18结合蛋白在骨髓增生异常综合征中的临床意义

**DOI:** 10.3760/cma.j.cn121090-20231009-00178

**Published:** 2024-03

**Authors:** 婷 王, 柠源 冉, 邱林 陈, 冬兰 刘, 梦桐 臧, 念滨 李, 欣 贺, 晶 关, 蓉 付, 宗鸿 邵

**Affiliations:** 1 天津医科大学总医院，天津 300052 Department of Hematology, General Hospital of Tianjin Medical University, Tianjin 300052, China; 2 天津市骨髓衰竭及癌性造血克隆防治重点实验室，天津 300052 Tianjin Key Laboratory of Bone Marrow Failure and Malignant Hemopoietic Clone Control, Tianjin 300052, China; 3 中南大学湘雅医学院附属常德医院（常德市第一人民医院）血液内科，常德 415003 Changde Hospital, Xiangya School of Medicine, Central South University（The First People's Hospital of Changde）, Changde 415003, China; 4 北京朝阳医院肾内科，北京 100020 Department of Nephrology, Beijing Chaoyang Hospital, Beijing 100020, China

**Keywords:** 骨髓增生异常综合征, IL-18, IL-18结合蛋白, 游离IL-18, Myelodysplastic syndrome, IL-18, IL-18-binding protein, Free IL-18

## Abstract

**目的:**

分析IL-18、IL-18结合蛋白（IL-18BP）在骨髓增生异常综合征（MDS）患者骨髓液中的表达水平及临床意义。

**方法:**

纳入2020年7月至2021年2月天津医科大学总医院血液科收治的初诊MDS患者43例，对照组包括急性髓系白血病（AML）患者14例、缺铁性贫血（IDA）患者25例，检测骨髓上清中IL-18和IL-18BP水平，分析其与MDS的严重程度、CD8^+^ T细胞及NK细胞功能的相关性。

**结果:**

MDS患者骨髓上清中IL-18、IL-18BP和游离IL-18（fIL-18）的水平均高于IDA组，fIL-18的水平与MDS-IPSS评分呈线性负相关。MDS患者CD8^+^ T细胞表面IL-18受体（IL-18Rα）表达低于IDA组，fIL-18及IL-18Rα水平均与MDS患者CD8^+^ T细胞功能呈正相关。

**结论:**

IL-18在MDS患者骨髓微环境中表达增加，但由于IL-18BP的存在导致fIL-18相对减少，与MDS疾病严重程度和CD8^+^ T细胞功能减低有关。

## Abstract (en)

**Objective:**

To analyze the level and clinical significance of IL-18 and IL-18-binding protein（BP）in the bone marrow of patients with myelodysplastic syndrome（MDS）.

**Methods:**

A total of 43 newly diagnosed patients with MDS who were admitted to the Department of Hematology, Tianjin Medical University General Hospital, from July 2020 to February 2021 were randomly selected. The control group consisted of 14 patients with acute myeloid leukemia（AML）and 25 patients with iron-deficiency anemia（IDA）. The levels of IL-18 and IL-18 BP in the bone marrow supernatant were measured, and their correlations with MDS severity, as well as the functionality of CD8^+^ T cells and natural killer cells, was analyzed.

**Results:**

The levels of IL-18, IL-18 BP, and free IL-18（fIL-18）in the bone marrow supernatant of patients with MDS were higher than in the IDA group. The level of fIL-18 was linearly and negatively correlated with the MDS- International Prognostic Scoring System（IPSS）score. IL-18 receptor（IL-18Rα）expression on CD8^+^ T cells in the MDS group was lower than in the IDA group, and the levels of fIL-18 and IL-18Rα were positively correlated with CD8^+^ T-cell function in the MDS group.

**Conclusion:**

IL-18 BP antagonizes IL-18, leading to a decrease in fIL-18 in the bone marrow microenvironment of patients with MDS, affecting CD8^+^ T-cell function, which is closely related to MDS severity; therefore, it may become a new target for MDS treatment.

骨髓增生异常综合征（MDS）是髓系克隆造血导致的血液系统恶性疾病，表现为血细胞减少、骨髓病态或无效造血，高风险向急性髓系白血病（AML）转化[1]。免疫机制在MDS的发病中发挥重要作用[2]。IL-18是一种多效性促炎细胞因子，参与先天性和适应性免疫反应的调节[3]。在 IL-12和（或）IL-15存在的情况下，IL-18诱导巨噬细胞、NK细胞、T细胞和B细胞产生干扰素γ（IFN-γ）；IL-18与IL-23结合可促进Th17细胞的生长[4]–[5]。在IL-18的刺激下NK细胞及T细胞抗肿瘤作用均增强[6]。研究显示IL-18在包括MDS在内的多种肿瘤患者中表达增高[7]，但给予IL-18治疗未能发挥预期抑制肿瘤生长的理想疗效[8]。IL-18结合蛋白（IL-18BP）是一种天然的游离免疫检查点蛋白，可竞争性抑制IL-18与其受体（IL-18Rɑ）结合，且与IL-18的亲和力远高于IL-18Rɑ。目前已发现IL-18BP在多种肿瘤[9]–[11]和自身免疫性疾病[12]–[13]中高表达，但在MDS患者中的作用尚不清楚。本研究检测了MDS患者骨髓上清中IL-18和IL-18BP的表达水平，探讨在MDS患者中检测的临床意义。

## 病例与方法

一、病例资料

回顾性分析2020年7月至2021年2月在天津医科大学总医院血液科确诊的43例初治MDS患者及对照病例［AML患者14例、缺铁性贫血（IDA）患者25例］。所有患者均完成骨髓穿刺、骨髓病理、染色体核型、组织化学分析等相关检查，排除感染、实体肿瘤及其他血液系统疾病。MDS患者符合2016年WHO诊断分型标准，AML患者符合2017年WHO诊断分型标准。该研究经天津医科大学总医院伦理委员会批准（批件号：IRB2019-210-01）。收集所有患者外周血及骨髓液3 ml到EDTA抗凝管中，室温下1 000×*g*离心15 min，吸取500 µl上清至1.5 ml无酶EP管中，密封在−80 °C冰箱中保存，备后期检测IL-18和IL-18BP的表达水平。

二、ELISA法检测骨髓上清中IL-18和IL-18BP的表达水平

本研究使用的人IL-18 ELISA试剂盒（最小检测限为20 pg/ml）、人IL-18BP试剂盒（最小检测限为47 pg/ml）均购自北京博奥森生物技术有限公司。使用酶标仪Elx800（美国Bio Tek公司产品）按照试剂盒说明进行检测操作。在测量每个样品IL-18和IL-18BP的浓度后，采用质量作用法[14]计算游离IL-18（fIL-18）的水平（即没有与其抑制剂IL-18BP结合的细胞因子的部分）。已知IL-18的相对分子质量为18.4×10^3^，IL-18BP的相对分子质量为17.6×10^3^，IL-18和IL-18BP按1∶1结合，其相互作用的Kd值为0.4 nmol/L。

三、流式细胞术检测CD8^+^ T细胞及NK细胞功能

CD3-PerCP、CD8-APC-CY7、IL-18Rα-PE、PD-1-PE-CY7、TIM-3-BV421、IFN-γ-FITC、Perforin-APC、Granzyme B-BV421用于检测CD8^+^T细胞的数量及功能；CD3-PerCP、CD56-FITC、IL-18Rα-PE、PD-1-PE-CY7和TIM-3-BV421、IFN-γ-FITC、Perforin-APC、Granzyme B-PE-CY7用于检测NK细胞的数量及功能。运用流式细胞仪CytoFLE进行检测，利用CytExpert进行数据分析。

四、统计学处理

采用SPSS 25.0统计软件和GraphPad Prism 5.01统计软件进行数据分析和图形处理。连续变量的正态性判定采用Shapiro-Wilk检验，若为正态分布则用*x±s*表示，两组间比较采用独立样本*t*检验，多组间的比较采用单因素方差分析。若为非正态分布则用*M*（*IQR*）表示，两组间比较采用Mann-Whitney *U*检验，多组间比较采用Kruskal-Wallis *H*秩和检验，进一步采用Dunn法进行多重比较。两个变量的相关性分析采用Pearson或Spearman法。双侧*P*<0.05为差异有统计学意义。

## 结果

一、MDS患者骨髓上清中IL-18和IL-18BP的表达水平

MDS患者和AML患者骨髓上清中IL-18表达水平分别为（211.30±157.20）ng/L和（202.90±102.40）ng/L，均明显高于IDA组的（84.97±64.78）ng/L（*P*值分别为<0.001、0.004）。MDS患者和AML患者的IL-18BP表达水平分别为（33 149±12 134）ng/L和（40 221±14 259）ng/L，均明显高于IDA组的（21 796±7 858）ng/L（*P*值分别为0.004、<0.001）。MDS患者和AML患者的fIL-18表达水平分别为（38.40±25.88）ng/L和（31.15±14.34）ng/L，MDS患者的fIL-18水平显著高于IDA组的（22.84±18.66）ng/L（*P*＝0.039）（图1），较AML组表达水平差异无统计学意义（*P*＝1.000）。

**图1:**
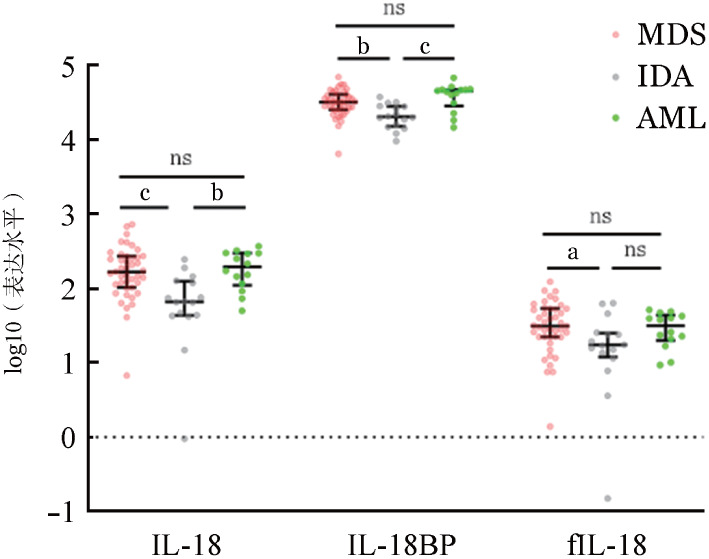
MDS、AML和IDA患者骨髓上清中IL-18、IL-18BP和游离IL-18的表达水平 注 MDS：骨髓增生异常综合征；AML：急性髓系白血病；IDA：缺铁性贫血；IL-18BP：IL-18结合蛋白；^ns^*P*>0.05、^a^*P*<0.05、^b^*P*<0.01、^c^*P*<0.001

为探究IL-18BP对IL-18的拮抗作用是否仅局限于骨髓原位，我们对31例MDS患者骨髓及外周血同时采样，分别检测IL-18、IL-18BP水平并进行Pearson相关性分析。结果显示MDS患者外周血与骨髓的IL-18（*r*＝0.935，*P*<0.001）、IL-18BP（*r*＝0.897，*P*<0.001）均呈正相关，提示二者具有很好的一致性（图2）。

**图2:**
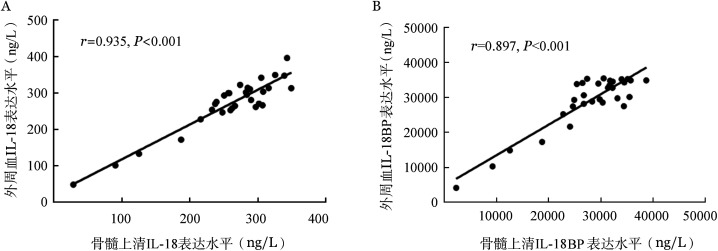
骨髓增生异常综合征（MDS）患者外周血及骨髓上清IL-18（A）和IL-18结合蛋白（B）Pearson相关性分析

二、fIL-18表达水平与MDS严重程度的相关性

根据修订版MDS国际预后评分系统（IPSS-R），将43例初治MDS患者分为相对高危组（>3.5分）和相对低危组（≤3.5分）。分析发现，相对低危组患者IL-18BP的表达水平与相对高危组比较差异无统计学意义［（32 351±12 178）ng/L对（33 723±12 321）ng/L，*P*＝0.932］。相对低危组患者的fIL-18的表达水平明显高于相对高危组［（50.26±30.15）ng/L对（29.85±18.61）ng/L，*P*＝0.016］（图3）。

**图3:**
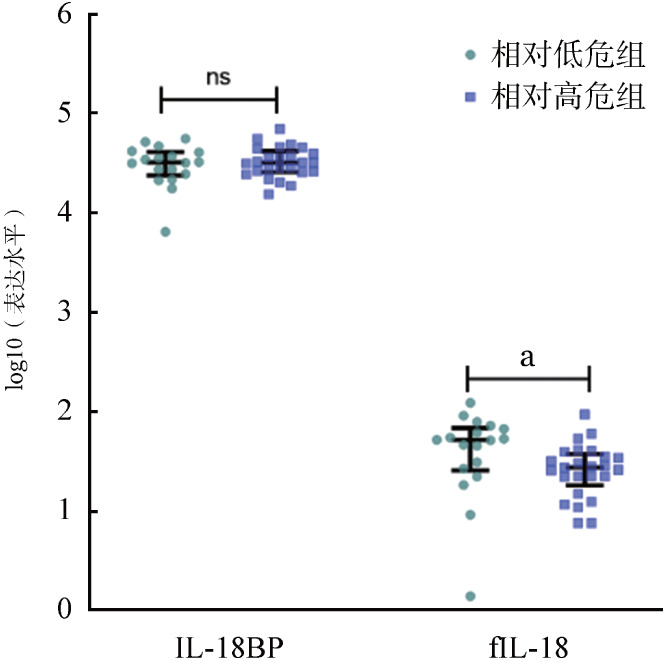
骨髓增生异常综合征（MDS）患者IPSS-R评分相对高危组和相对低危组骨髓上清中IL-18BP和fIL-18的表达水平 注 IPSS-R：修订版MDS国际预后评分系统；IL-18BP：IL-18结合蛋白；fIL-18：游离IL-18；^ns^*P*>0.05、^a^*P*<0.05

骨髓上清中fIL-18与骨髓原始细胞比例（*r*＝−0.36，*P*＝0.03）、国际预后积分系统（IPSS）评分（*r*＝−0.35，*P*＝0.02）、IPSS-R评分（*r*＝−0.38，*P*＝0.01）和WHO分型预后积分系统（WPSS）评分（*r*＝−0.31，*P*＝0.04）均呈负相关。

在与突变基因相关性的研究结果显示：DTA（DNMT3A、TET2、ASXL1）基因突变与IL-18BP水平呈正相关（*r*＝0.412，*P*＝0.008），但与IL-18水平（*r*＝0.18，*P*＝0.25）、fIL-18（*r*＝−0.07，*P*＝0.65）无明显线性相关性。剪切子相关基因突变（SF3B1、U2AF1、SRSF2和ZRSR2）与IL-18（*r*＝0.15，*P*＝0.34）、IL18BP（*r*＝0.09，*P*＝0.60）、fIL-18（*r*＝0.16，*P*＝0.33）均水平无明显线性相关性。

三、fIL-18表达水平对MDS患者CD8^+^ T细胞功能的影响

1. MDS患者CD8^+^ T细胞功能：MDS患者和AML患者CD8^+^ T细胞分泌穿孔素水平分别为（12.35±9.71）％和（7.80±4.10）％，均显著低于IDA组的（22.25±13.66）％（*P*值分别为0.040、0.001）。MDS与AML患者骨髓CD8^+^ T细胞分泌的穿孔素水平差异无统计学意义（*P*＝0.622）。MDS患者和AML患者骨髓CD8^+^ T细胞分泌的IFN-γ水平分别为（16.65±15.11）％和（11.13±9.31）％，明显低于IDA组的（29.17±17.11）％（*P*值分别为0.027、0.002）（图4）。MDS、AML和IDA组患者骨髓CD8^+^ T细胞分泌的颗粒酶B水平分别为（38.46±15.40）％、（39.81±14.31）％和（43.94±18.97）％，组间差异无统计学意义（*P*＝1.000）。

2. MDS患者PD-1^+^TIM-3^+^CD8^+^/CD8^+^ T细胞比例：MDS及AML患者PD-1^+^CD8^+^/CD8^+^细胞比例分别为（43.44±16.17）％和（40.73±23.30）％，均显著高于IDA组的（22.63±11.91）％（*P*值分别为<0.001、0.020）。MDS和AML患者TIM-3^+^CD8^+^/CD8^+^ T细胞比例分别为（42.12±19.51）％和（38.16±19.45）％，均显著高于IDA组的（20.77±12.70）％（*P*值分别为<0.001、0.031）。MDS和AML患者的PD-1^+^TIM-3^+^CD8^+^/ CD8^+^ T细胞比例分别为（17.55±11.57）％和（16.21±15.75）％，均显著高于IDA组的（6.78±4.90）％（*P*值分别为<0.001、0.013），提示MDS和AML患者CD8^+^ T细胞存在免疫耗竭状态（图5）。

3. MDS患者T细胞上IL-18Rα的表达：MDS患者和AML患者CD8^+^ T细胞上IL-18Rα的表达水平分别为（57.44±15.37）％和（55.94±13.32）％，均显著低于IDA组的（69.32±14.98）％（*P*值分别为0.036、0.048），MDS和AML患者之间差异无统计学意义（*P*＝1.000）（图4）。

**图4:**
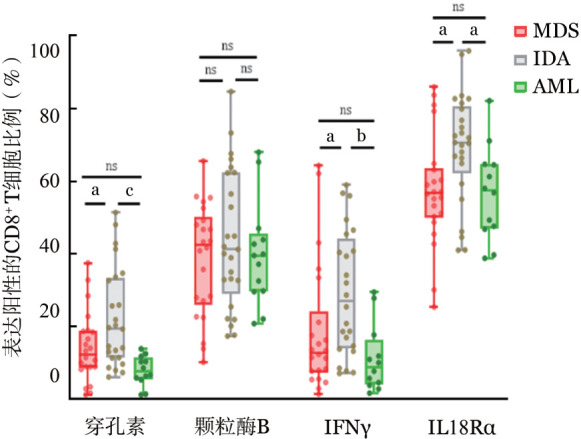
MDS、AML及IDA患者中CD8^+^ T细胞功能及IL-18Rα的表达 注 MDS：骨髓增生异常综合征；AML：急性髓系白血病；IDA：缺铁性贫血；IL-18Rα：IL-18受体；^ns^*P*>0.05，^a^*P*<0.05，^b^*P*<0.01，^c^*P*<0.001

**图5:**
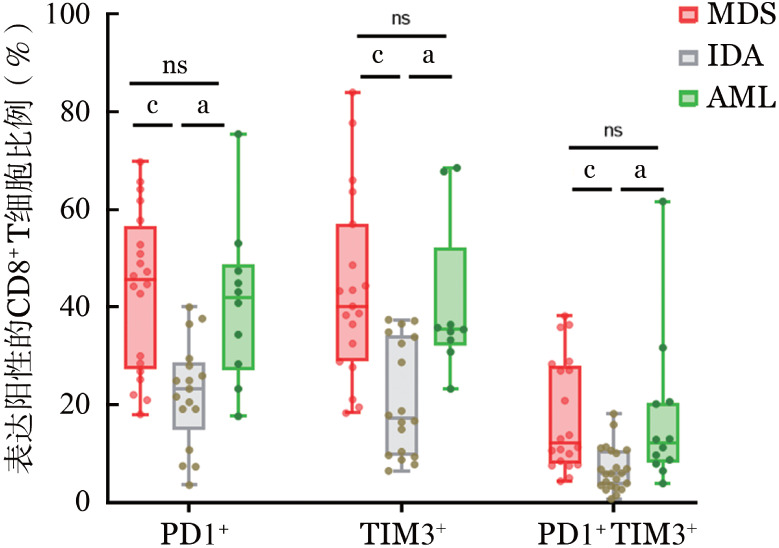
骨髓增生异常综合征（MDS）患者骨髓中CD8^+^ T细胞表面免疫检查点的表达水平（^ns^*P*>0.05，^a^*P*<0.05，^c^*P*<0.001）

4. MDS患者的fIL-18和IL-18Rα与CD8^+^ T细胞免疫状态的相关性：MDS患者骨髓上清中fIL-18表达水平与CD8^+^ T细胞分泌的穿孔素（*r*＝0.54，*P*＝0.04）和IFN-γ（*r*＝0.64，*P*＝0.01）呈正相关，与颗粒酶B无明显线性相关性（*r*＝0.29，*P*＝0.30）；CD8^+^ T细胞表面的IL-18Rα与穿孔素、颗粒酶B及IFN-γ表达水平均呈正相关（*r*值分别为0.60、0.53、0.52，*P*值分别为0.02、0.04、0.03）。但MDS患者骨髓上清中的IL-18BP水平与穿孔素、颗粒酶B及IFN-γ表达水平均无明显线性相关性（*r*值分别为0.01、0.09、−0.29，*P*值分别为0.96、0.74、0.29）。本研究并未发现MDS患者骨髓上清中IL-18BP、fIL-18及IL-18Rα与PD-1^+^TIM-3^+^CD8^+^ T细胞比例的线性相关性，提示IL-18或fIL-18水平可能与CD8^+^ T细胞耗竭无显著关联。

四、fIL-18表达水平对MDS患者NK细胞功能的影响

1. MDS患者NK细胞分泌的穿孔素和颗粒酶B的表达水平：MDS患者骨髓NK细胞分泌的穿孔素水平显著低于IDA组［（57.36±23.81）％对（78.21±12.36）％，*P*＝0.042］，颗粒酶B水平明显低于IDA组［（57.44±15.37）％对（69.32±14.98）％，*P*＝0.034］，骨髓NK细胞上IL-18Rα的表达水平显著低于IDA组［（63.61±20.40）％对（81.30±11.10）％，*P*＝0.040］（图6）。

**图6:**
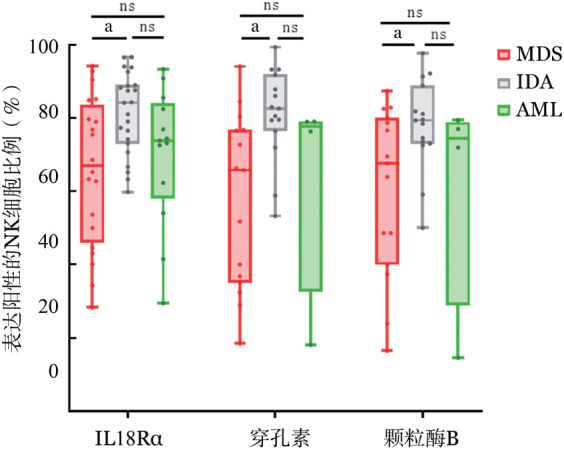
骨髓增生异常综合征（MDS）患者骨髓CD8^+^ T细胞和NK细胞的功能（^ns^*P*>0.05、^a^*P*<0.05）

2. MDS患者IL-18BP和IL-18Rα与NK细胞免疫状态的相关性：MDS患者骨髓上清中的fIL-18（*r*＝0.07，*P*＝0.83）、IL-18BP（*r*＝0.08，*P*＝0.80）、IL-18Rα（*r*＝0.07，*P*＝0.82）表达水平与骨髓NK细胞分泌的穿孔素无明显线性相关性；fIL-18（*r*＝0.14，*P*＝0.66）、IL-18BP（*r*＝0.16，*P*＝0.61）、IL-18Rα（*r*＝−0.21，*P*＝0.50）表达水平与骨髓NK细胞分泌的颗粒酶B无明显线性相关性。

## 讨论

MDS是造血干细胞的一种恶性克隆性疾病，以无效和病态造血为特征，可转变为AML。MDS的发病机制涉及许多因素，包括细胞遗传学变化和分子异常，如基因突变和表观遗传学变化，以及细胞免疫和免疫微环境的紊乱等。

IL-18是一种促炎症和免疫调节因子。在一些临床前模型中已经发现IL-18具有抗肿瘤活性[15]–[16]。重组IL-18（rIL-18）通过激活CD4^+^、CD8^+^ T细胞和（或）NK细胞介导的反应，可消除小鼠黑色素瘤或肉瘤[17]；在小鼠模型中，rIL-18与免疫检查点抑制剂（ICI）和嵌合抗原受体T细胞（CAR-T细胞）具有协同作用[18]。尽管在动物实验中显示出显著疗效，但IL-18在临床应用上却未见明显有效性[19]，这引起广泛研究者的困惑。这种困惑持续到人们发现了IL-18BP。很多研究表明，肿瘤微环境中不同因素介导了IL-18BP的表达，IL-18BP限制了IL-18与其受体的结合，从而降低IL-18的生物活性。这一发现揭示了肿瘤通过IL-18BP表达来逃避免疫反应的潜在机制，对肿瘤治疗研究具有重要意义。

本研究发现，MDS患者骨髓上清中IL-18表达水平明显升高，这说明MDS体内存在抗肿瘤的炎症反应，但我们同样发现IL-18BP表达水平也同步升高，这就抑制了IL-18抗肿瘤作用。根据质量作用定律[14]和骨髓上清中IL-18和IL-18BP的表达水平，我们计算了MDS患者骨髓fIL-18的表达水平，发现MDS患者fIL-18的表达水平仍高于IDA组，并且fIL-18在MDS中高于AML，说明在MDS阶段，机体的有效炎症反应强于AML。本研究还发现不同分期的MDS患者骨髓fIL-18的表达水平也不相同，相对低危组MDS患者fIL-18的表达水平高于相对高危组。

肿瘤患者的CD8^+^ T细胞及NK细胞不仅发挥免疫监视作用，而且是机体杀灭肿瘤细胞最主要的武器。本研究再次验证了MDS患者CD8^+^ T细胞及NK细胞分泌功能分子（如穿孔蛋白、颗粒酶、IFN-γ）的能力减低。且由于CD8^+^ T细胞及NK细胞表面IL-18Rα表达减少，导致fIL-18促进CD8^+^ T及NK细胞活化能力减弱。MDS患者骨髓CD8^+^ T细胞存在耗竭状态，MDS患者的PD-1^+^TIM-3^+^CD8^+^ T细胞比例增高，但本研究并未发现IL-18BP、fIL-18以及IL-18Rα与骨髓CD8^+^ T细胞表面PD-1和TIM-3的表达水平的相关性，说明IL-18通过调节IL-18Rα调控T细胞的功能，可能并未参与CD8^+^ T细胞耗竭的过程。

近期，Zhou等[8]创造了一种诱饵受体DR-18，可以有效地防止IL-18被IL-18BP中和，但不影响IL-18与IL-18受体结合，介导下游的信号转导。该药物目前正在进行临床开发，未来有望用于多种肿瘤性疾病的治疗。本实验证实MDS患者骨髓上清中IL-18的表达水平虽然不低，但由于IL-18BP的存在导致fIL-18表达水平相对不足，加之MDS患者CD8^+^ T和NK细胞表面IL-18Rα的下调，导致CD8^+^ T和NK细胞炎症反应能力不足，抗肿瘤功能受抑。因此，IL-18BP拮抗剂和（或）促进IL-18受体上调的药物，有望成为治疗MDS的新选择。
